# Supplementary material for: Ultrafast complete dechlorination enabled by ferrous oxide/graphene oxide catalytic membranes via nanoconfinement advanced reduction
Source: Nat Commun. 2024 Nov 7;15:9607. doi: 10.1038/s41467-024-54055-x (PMC11541988; doi:10.1038/s41467-024-54055-x)
Supplement: Supplementary file 1 — Supplementary Information [file 41467_2024_54055_MOESM1_ESM.pdf]

Supplementary Information

Ultrafast complete dechlorination enabled by ferrous oxide/graphene oxide catalytic membranes via nanoconfinement advanced reduction

Qian Xiao[1,2,3], Wanbin Li[4*], Shujie Xie[1], Li Wang[1], Chuyang Y. Tang[1,5*]

[1]Department of Civil Engineering, The University of Hong Kong, Hong Kong, SAR 999077, China

[2]State Key Laboratory of Pollution Control and Resource Reuse, College of Environmental Science and Engineering, Tongji University, Shanghai, 200092, China

[3]Shanghai Institute of Pollution Control and Ecological Security, Shanghai, 200092, China

[4]Guangdong Key Laboratory of Environmental Pollution and Health, College of Environment and Climate, Jinan University, Guangzhou, 511443, China

[5]Materials Innovation Institute for Life Sciences and Energy (MILES), HKU-SIRI, Shenzhen, 518000, China

*Corresponding author. Email: tangc@hku.hk (C.T.), gandeylin@126.com (W.L.)

This file includes:

Supplementary Notes

Supplementary Figures 1 to 19

Supplementary Tables 1 to 3

Supplementary References 1 to 26

Supplementary Notes

Calculation of molar ratios of Fe to sulfite and Fe to DCAA

In this study, the $Fe_{c1.5}$/GO membrane had C, O, and Fe atomic contents of 75.98%, 23.27, and 0.75%, respectively. The Fe mass ratio of $Fe_{c1.5}$/GO was 0.0326 g g^{-1}. Based on the mass values in entire membrane system (including DCAA/sulfite solution and $Fe_{c1.5}$/GO membrane), the nominal molar ratios were calculated as 0.000234 for Fe to sulfite and 0.168 for Fe to DCAA (Supplementary Table 3). However, for membrane catalysis, the catalyst and reactants are confined in the transport channels of membranes. Therefore, the effective molar ratios in confined reaction regions of membranes are much higher than the nominal molar ratios in entire membrane system and those for catalysis in bulk solution[1-4]. As demonstrated in previous study[5], the GO membranes could adsorb water from aqueous solutions with ratios of 1–4 g g^{-1}. Considering (a) the sulfite concentration of 1 mM and the sulfite rejection of 50% by the $Fe_{c1.5}$/GO membrane and (b) the DCAA concertation of 180 μg L^{-1} and the limited rejection of membrane for DCAA (<2%), we could calculate that their effective concentrations in the reaction region are estimated to be 0.5 mM and 180 μg L^{-1}, respectively (see footnotes c and d of Supplementary Table 3). Based on the contents of sulfite, DCAA, and Fe within the catalytic membrane, the effective molar ratios of Fe to sulfite and Fe to DCAA in the reaction region are estimated to be 1168–292 and 418963–104741, respectively (Supplementary Table 3). The enriched ratios of Fe to sulfite and Fe to DCAA at confined interlayer spaces of membranes are beneficial for enhancing catalytic performance.

Supplementary Discussion

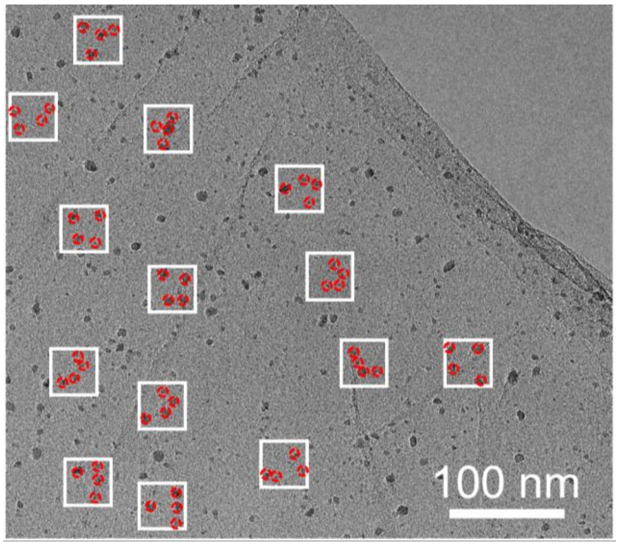

Supplementary Fig. 1. TEM images of $Fe_{c1.5}$/GO composites. Red circles indicate nanoparticles, and white squares represent 40 nm × 40 nm areas.

Additional analysis: The particle number density is almost the same of 4 per 40 nm × 40 nm for $Fe_{c1.5}$/GO composites, indicating that the nucleation occurs at GO surface.

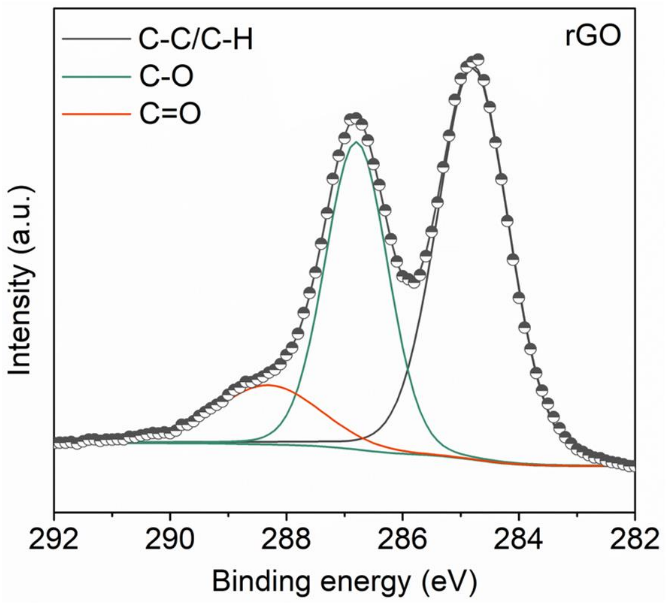

Supplementary Fig. 2. C *1s* XPS spectra of rGO. The C-C/C-H, C-O, and C=O are marked by grey, green, and orange, respectively.

Additional analysis: This finding suggests that the absence of iron results in a lower reduction.

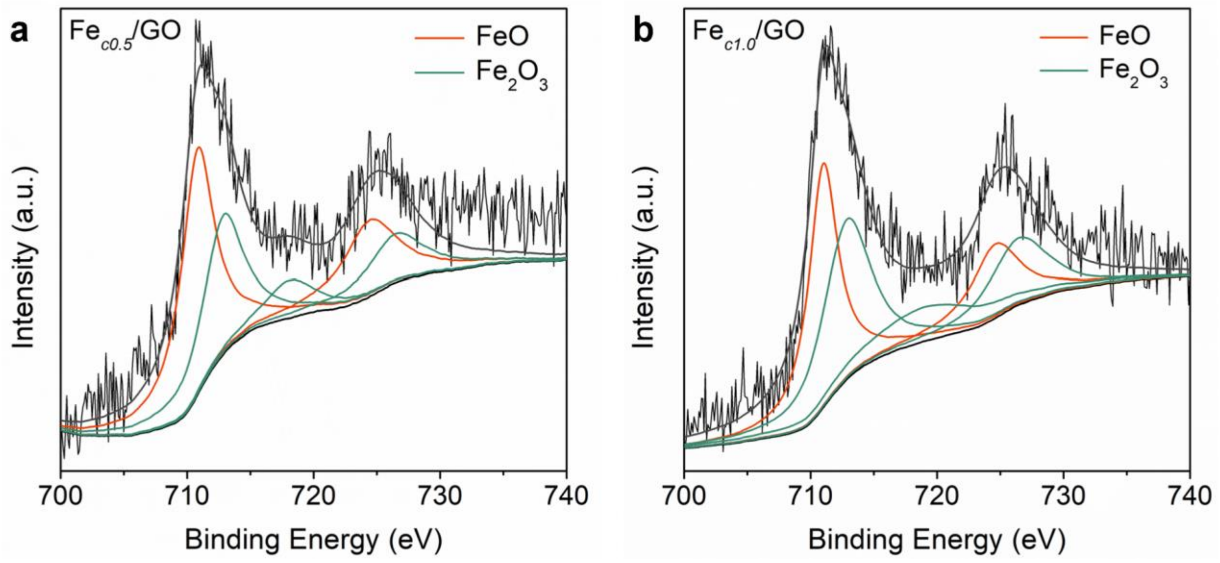

Supplementary Fig. 3. Fe *2p* XPS spectra of (**a**) $Fe_{c0.5}$/GO and (**b**) $Fe_{c1.0}$/GO. The FeO and Fe_2O_3 are marked by orange and green, respectively.

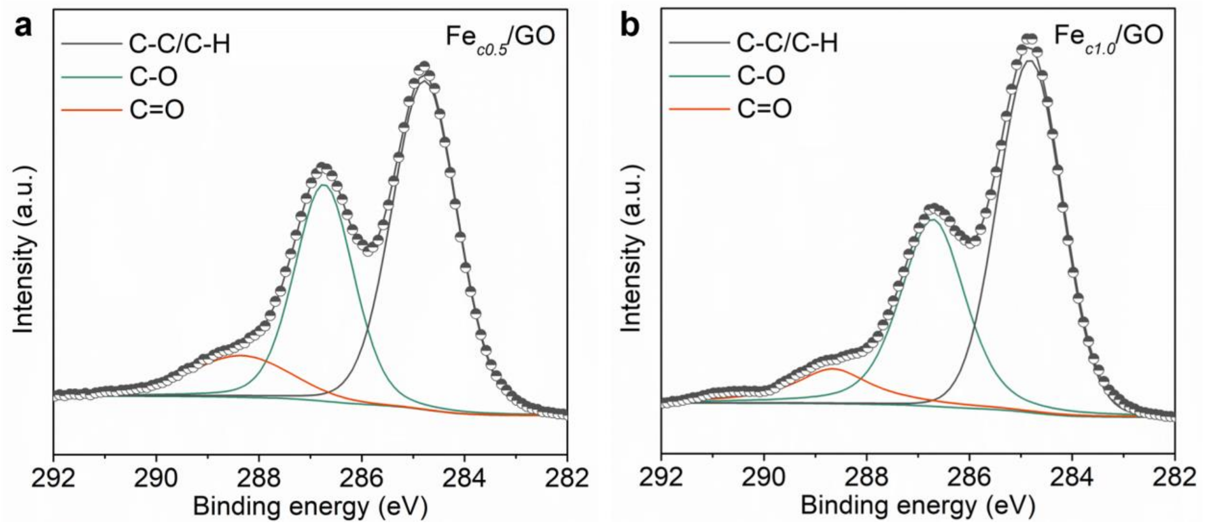

Supplementary Fig. 4. C *1s* XPS spectra of (**a**) Fe$_{c0.5}$/GO and (**b**) Fe$_{c1.0}$/GO. The C-C/C-H, C-O, and C=O are marked by grey, green, and orange, respectively.

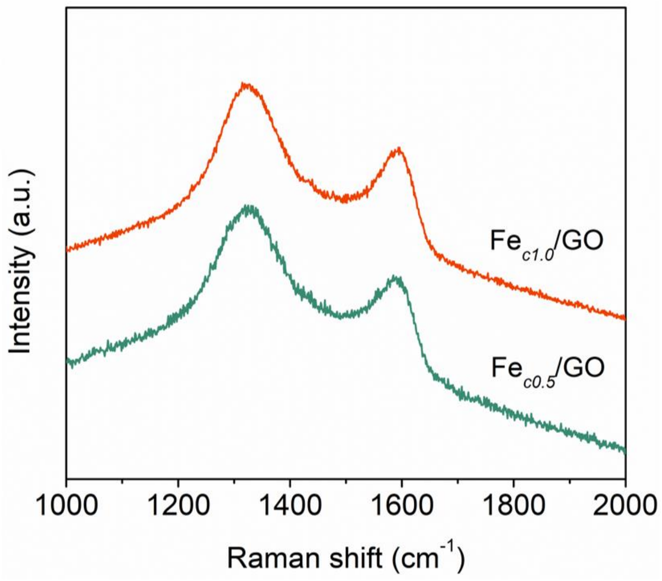

Supplementary Fig. 5. Raman spectra of Fe$_{c0.5}$/GO and Fe$_{c1.0}$/GO.

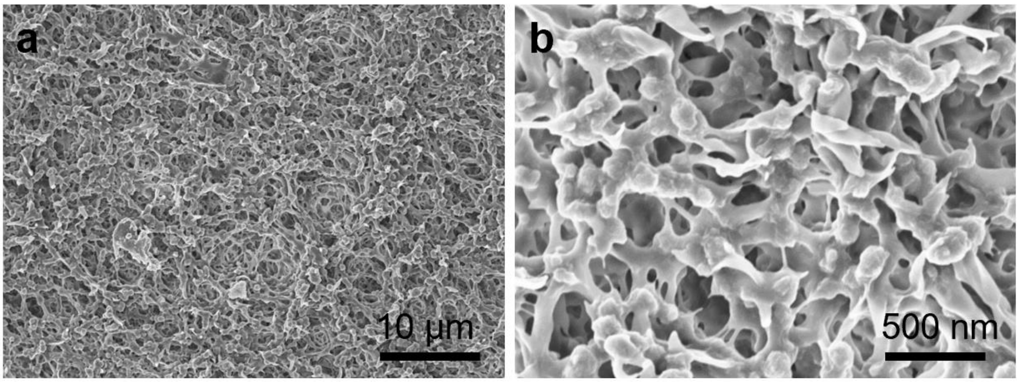

Supplementary Fig. 6. a,**b**, Plane-view SEM images of the polyvinylidene difluoride (PVDF) substrate.

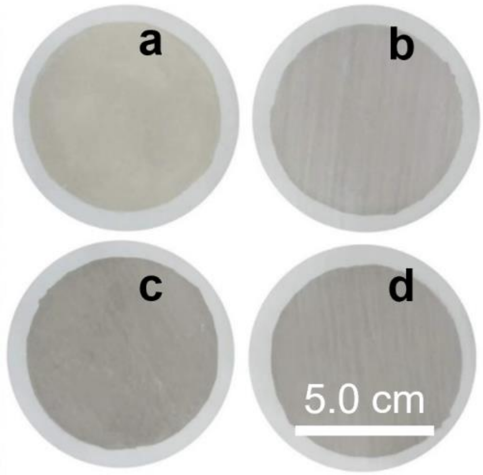

Supplementary Fig. 7. Photographs of Fe/GO membranes. **a, b, c**, and **d** represent GO, $Fe_{c0.5}$/GO, $Fe_{c1.0}$/GO, and $Fe_{c1.5}$/GO membranes, respectively, which indicated the iron loading amount of 0, 0.5, 1.0, and 1.5 mmol L^{-1} onto the GO surface, separately.

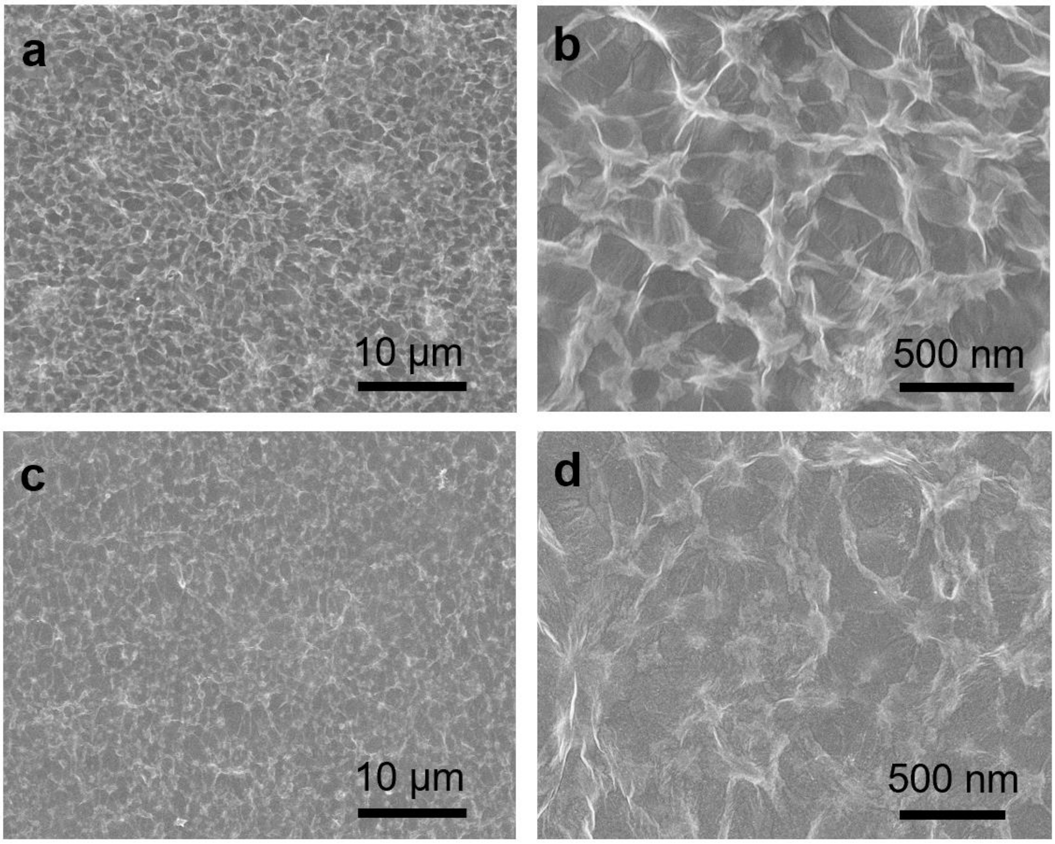

Supplementary Fig. 8. SEM images of (**a** and **b**) $Fe_{c0.5}$/GO and (**c** and **d**) $Fe_{c1.0}$/GO membranes.

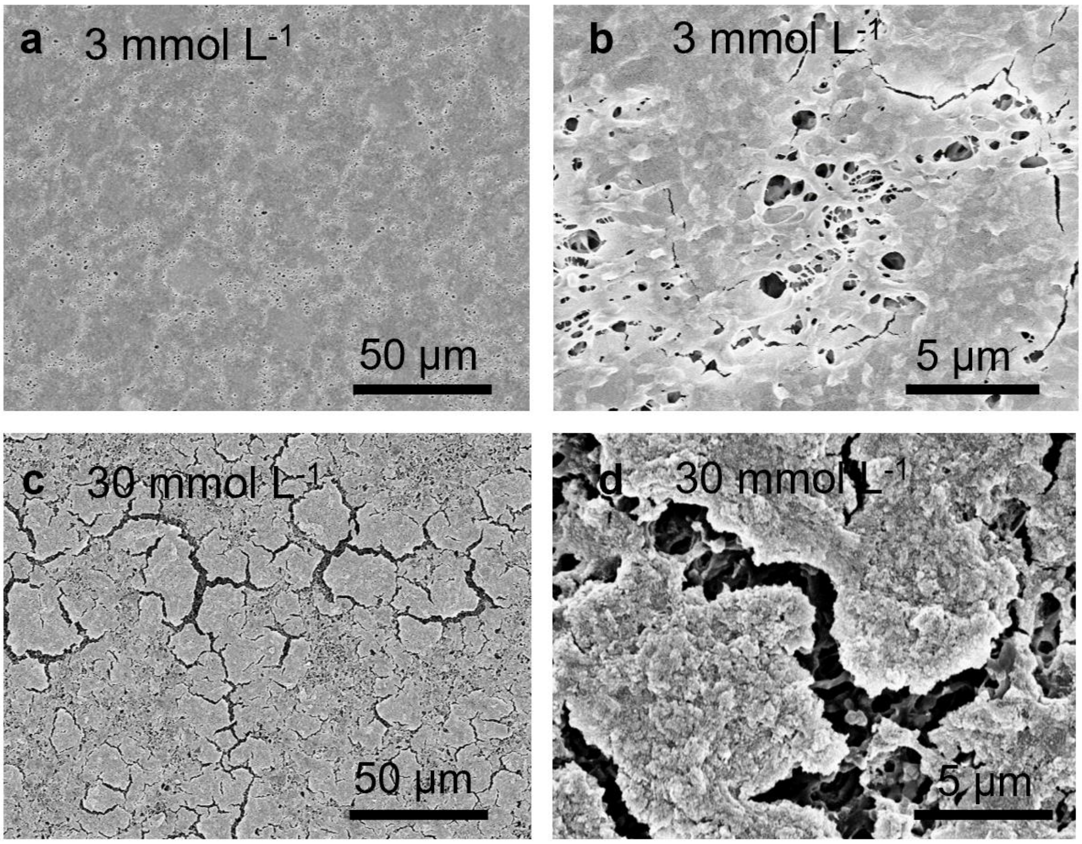

Supplementary Fig. 9. Plane-view SEM images of Fe/GO membranes by loading iron amounts of (**a** and **b**) 3 mmol L^{-1} and (**c** and **d**) 30 mmol L^{-1}. Notably, Supplementary Fig. 9b,d are enlarged sections of Supplementary Fig. 9a,c respectively.

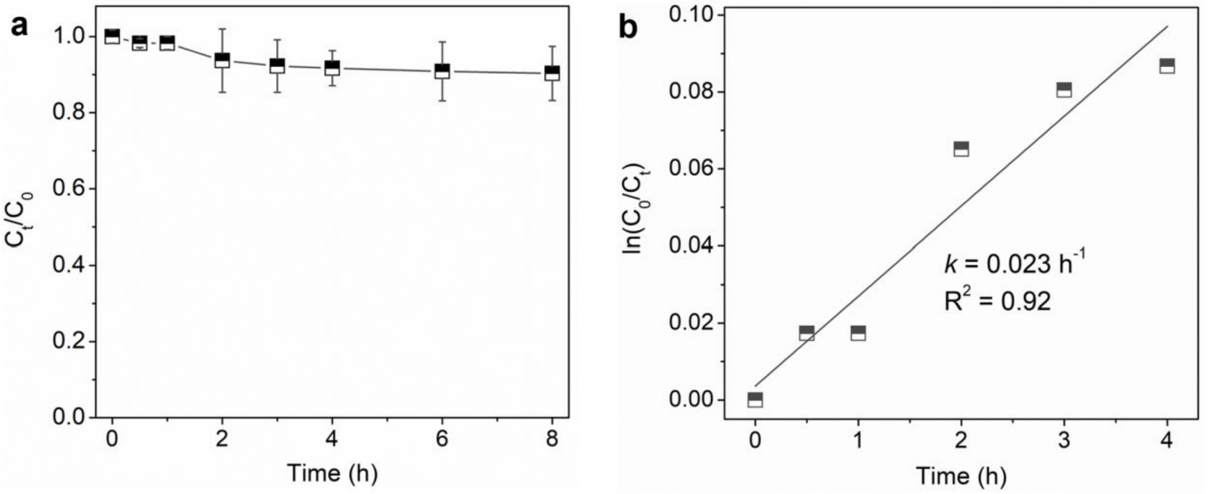

Supplementary Fig. 10. a, Reduction of DCAA over reaction time in $Fe_{c1.5}$/GO composites/S(IV) systems. **b**, The $\ln(C_0/C_t)$ values versus reaction time and the fitting by a pseudo-first-order removal model in $Fe_{c1.5}$/GO composites/S(IV) bulk solutions. Conditions: initial DCAA levels = 180 μg L^{-1}, $Fe_{c1.5}$/GO = 0.5 g L^{-1}, initial S(IV) levels = 1.0 mM, $pH_{ini.}$ 7.0 ± 0.1, and 25 ± 0.5 ^oC. Error bars represent the standard deviation from at least triplicate experiments. Some of the error bars are smaller than the symbols.

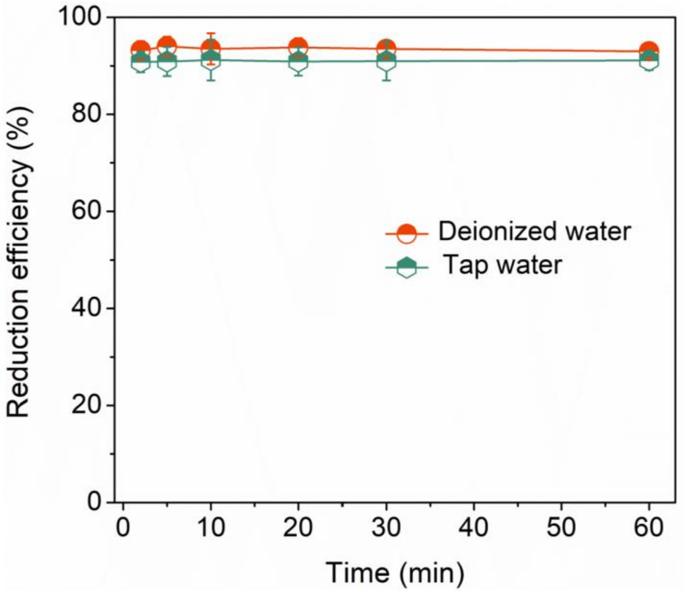

Supplementary Fig. 11. Reduction of DCAA in deionized water and tap water. Conditions of the feed solution: initial levels of DCAA = 80 μg L^{-1}, initial S(IV) levels = 1.0 mM, $pH_{ini.}$ 7.0 ± 0.1, and 25 ± 0.5 ^{o}C. Error bars represent the standard deviation from at least triplicate experiments. Some of the error bars are smaller than the symbols.

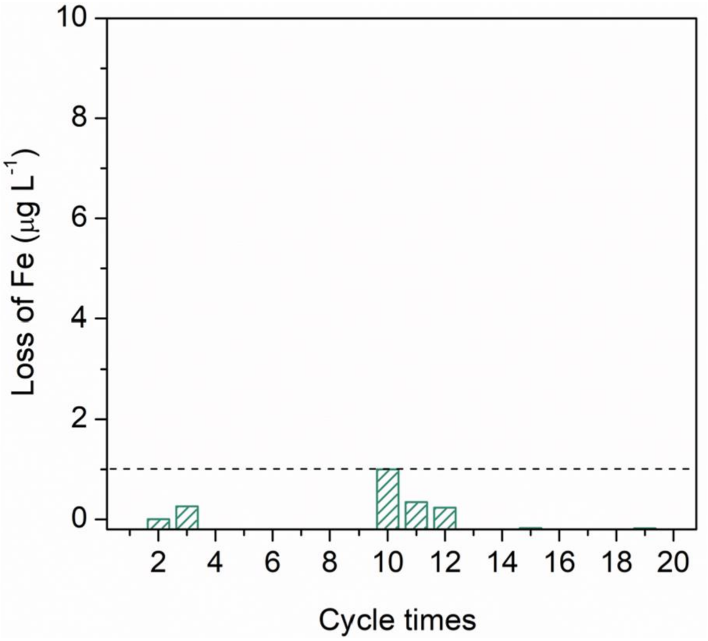

Supplementary Fig. 12. Loss of Fe of the Fe/GO membranes as a function of filtration cycles, with each cycle being operated for 1 hour. The dashed line represents the detection limit for Fe (i.e., 1.0 μg L^{-1}).

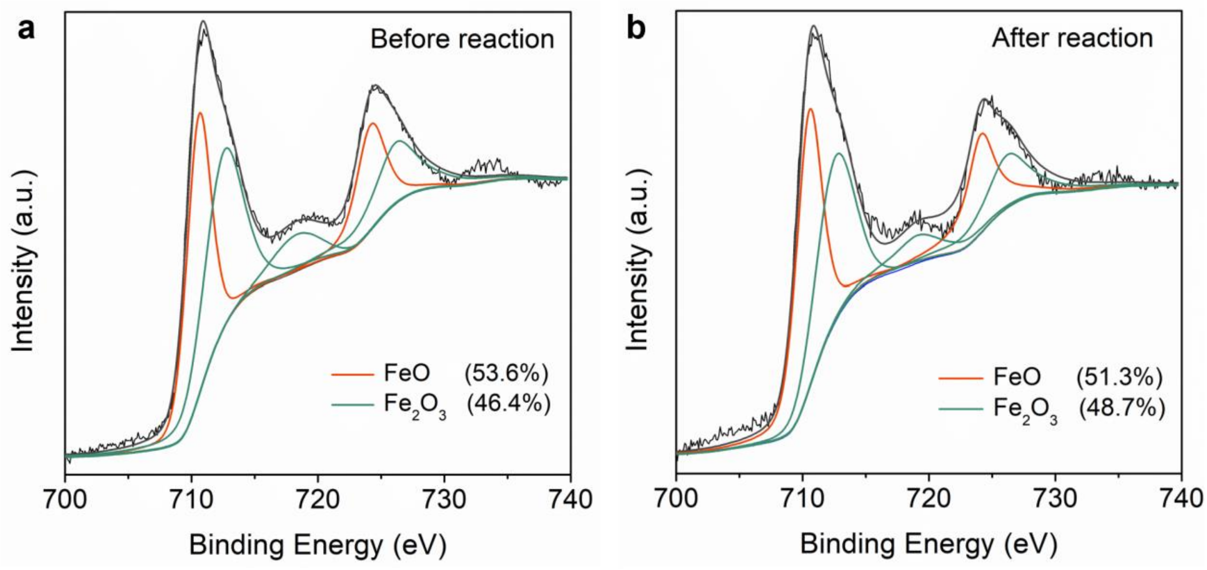

Supplementary Fig. 13. XPS analysis of Fe/GO membranes (**a**) before and (**b**) after the stability test. The FeO and Fe_2O_3 are marked by orange and green, respectively. The proportions of Fe(II) and Fe(III) were estimated by calculating the ratio of their respective peak areas to the total peak area of iron. These values have been added to the figures for better clarity.

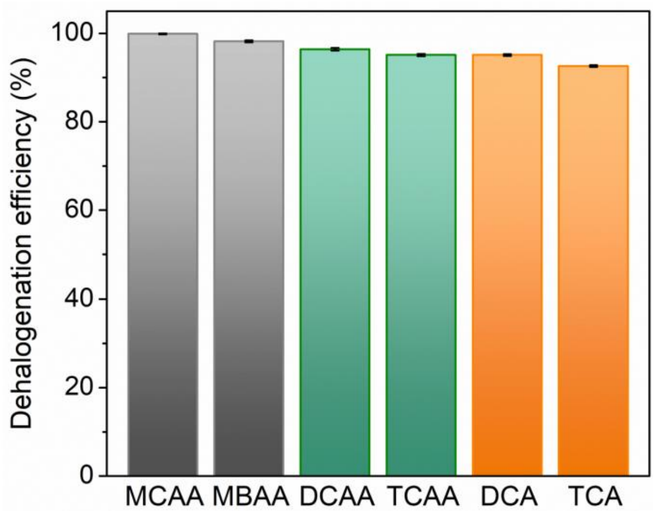

Supplementary Fig. 14. The dehalogenation efficiency in the permeate water after 60 min during the degradation of four HAAs and two chlorinated organic pollutants in water. Conditions of the feed solution for all membrane test: initial levels of each HAA = 180 μg L^{-1}, initial levels of DCA and TCA = 0.5 mM, initial S(IV) levels = 1.0 mM, $pH_{ini.}$ 7.0 ± 0.1, and 25 ± 0.5 ^{o}C. Error bars represent the standard deviation from at least triplicate experiments. Some of the error bars are smaller than the symbols.

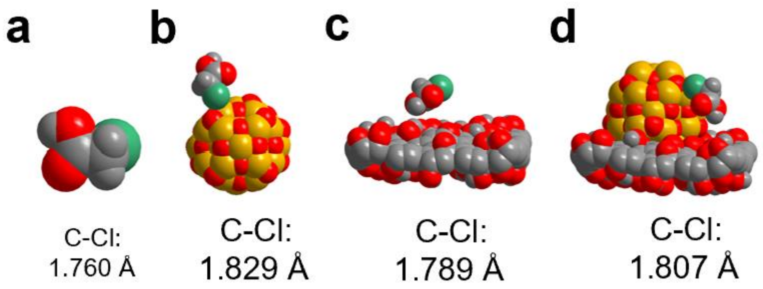

Supplementary Fig. 15. C-Cl length of DCAA (**a**) in a free mode and in the contact modes with (**b**) FeO, (**c**) GO, and (**d**) Fe/GO.

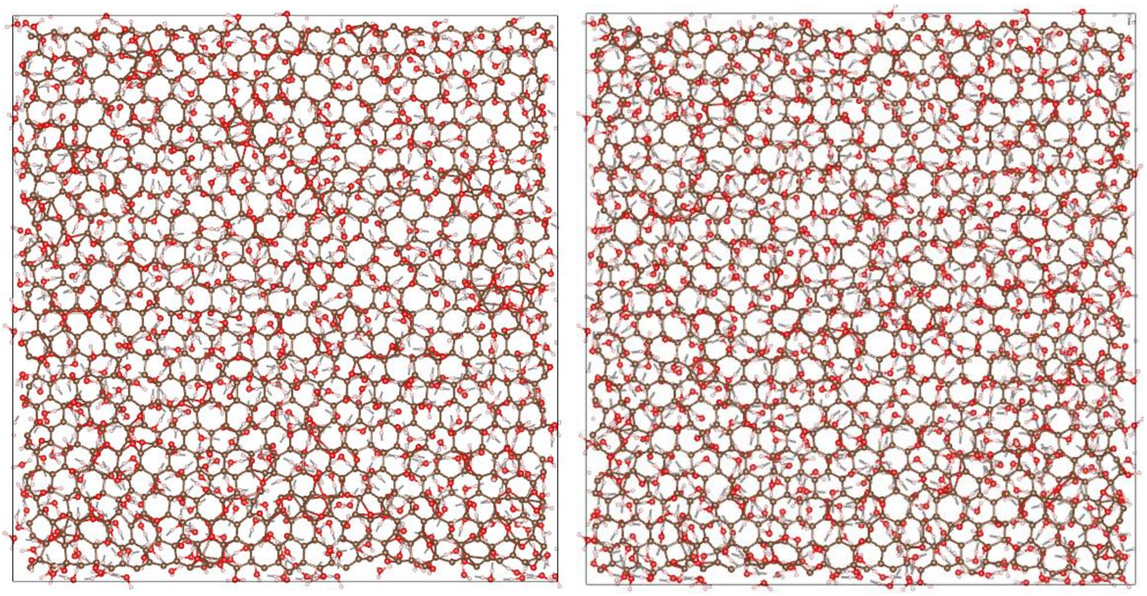

Supplementary Fig. 16. Molecular dynamic (MD) simulations of H_2O diffusion in GO (left) and rGO membranes (right). Oxygen atom, hydrogen atom, and carbon atom are marked by red, pink, and grey, respectively. H_2O molecules were homogeneously distributed in GO and rGO membranes.

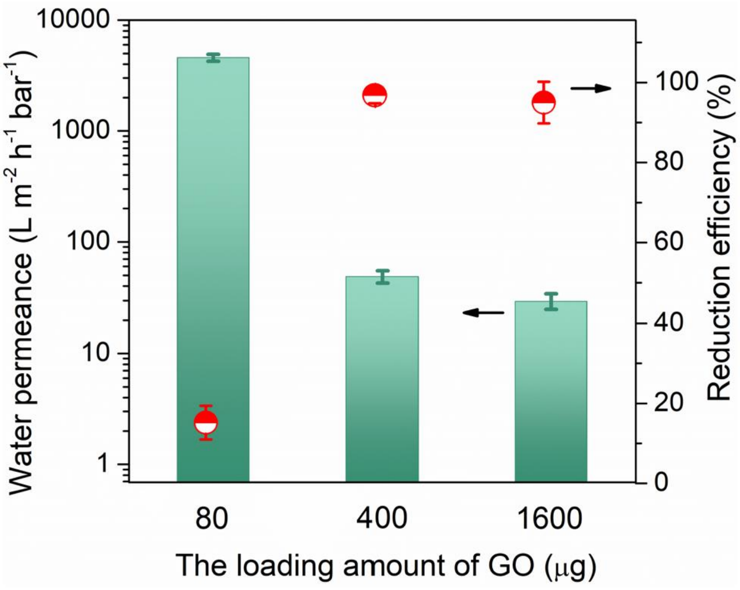

Supplementary Fig. 17. Water permeance and DCAA reduction efficiency of Fe/GO membranes as a function of the loading amount of GO. For instance, 400 µg GO is obtained by vacuum-filtration of 20 mL of a 20 mg L^{-1} GO solution that was loaded with Fe NPs onto the substrate membrane. Experimental conditions: initial DCAA level = 180 µg L^{-1}, initial S(IV) level = 1.0 mM, $pH_{ini.}$ 7.0 ± 0.1, applied pressure = 1.0 bar, membrane area = 8 cm^2, and temperature = 25 ± 0.5 °C. Error bars represent the standard deviation from at least triplicate experiments. Some of the error bars are smaller than the symbols.

Additional analysis: The volumetric loading of feed solution per hour can be obtained by the product of water permeance, applied pressure, and membrane area. In the current study, the volumetric loadings of feed solution within a filtration duration of 1 hr are 3.67 L, 0.0389 L, and 0.0236 L at GO loadings of 80 µg, 400 µg, and 1600 µg, respectively.

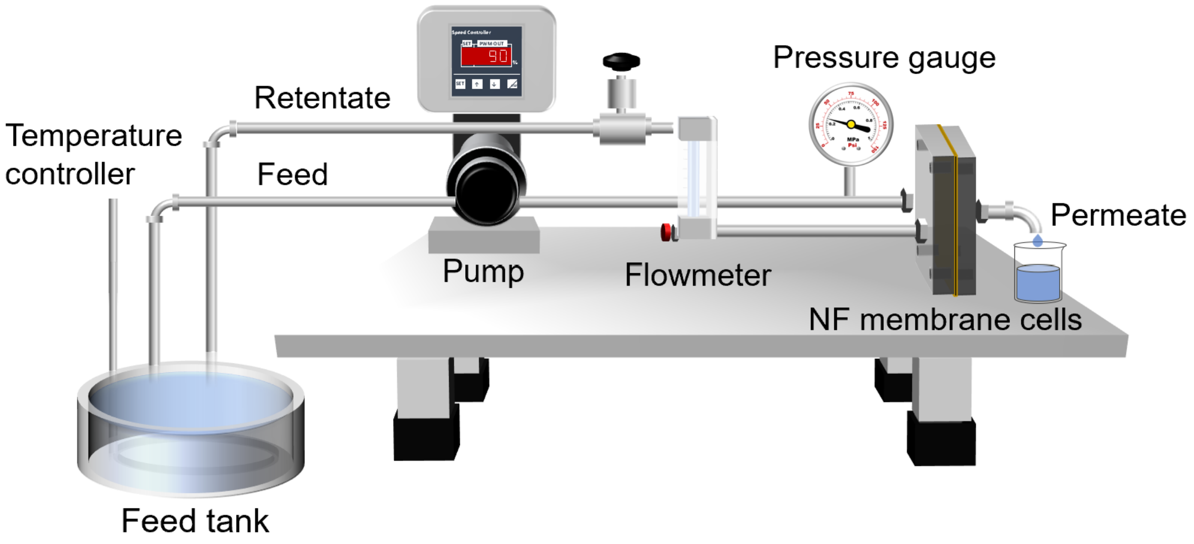

Supplementary Fig. 18. Schematic of the membrane catalysis by using the Fe/GO catalytic membrane. The filtration setup includes feed tank, pump, flowmeter, membrane cells, pressure gauge, and temperature controller. The filtration setup allows two possible operation modes: (1) recirculation mode for which only the retentate water recirculated back to the feed tank, while the permeate water underwent only one pass through the catalytic membrane and (2) one-pass mode for which both the retentate and permeate water are dumped without recirculation. Unless specified otherwise, all the catalytical filtration tests were performed in the recirculation mode to reduce the amount of feed solution needed. To ensure this recirculation does not affect the removal performance, additional one-pass catalytical filtration tests were also included for comparison (Supplementary Fig. 19).

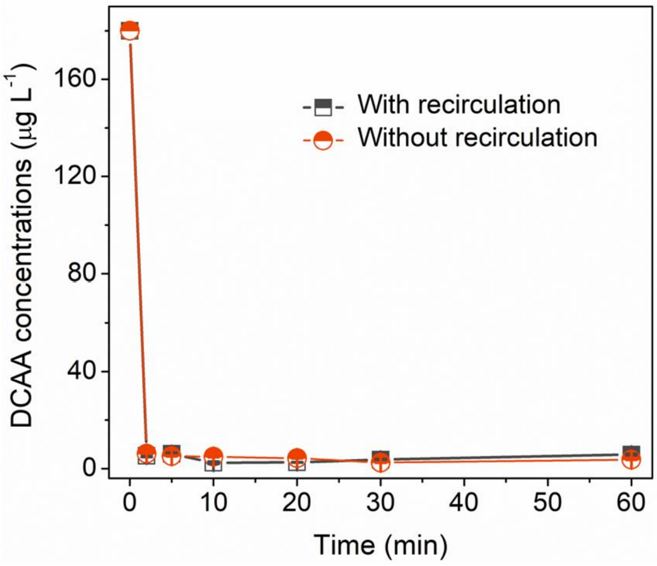

Supplementary Fig. 19. Removal of DCAA in the permeate water over time in Fe/GO membranes/S(IV) systems with and without recirculation. Conditions: initial DCAA levels = 180 μg L^{-1}, initial S(IV) levels = 1.0 mM, $pH_{ini.}$ 7.0 ± 0.1, and 25 ± 0.5 ^{o}C. Error bars represent the standard deviation from at least triplicate experiments. Some of the error bars are smaller than the symbols.

Additional analysis: In practice, a single pass treatment without recirculation is generally preferred. However, since the similar results obtained with and without recirculation, the current study performed the bench-scale tests predominantly with recirculation to reduce the amount of feed solution needed.

Supplementary Table 1. First-order rate constants and corresponding experimental conditions for HAAs removal in this study and previous studies.

No.	Processes	Types of HAAs	Conc. (μg L^{-1})	Reaction time (min)	Rate (min^{-1})	Remarks	Ref.
1	Chemical reduction in bulk solutions	BDCAA	29,120	15	0.177	13.9 g L^{-1} Fe^0	6
2		CDBAA	20,800	120	0.024		6
3		TBAA	29,700	120	0.024		6
4		TCAA	32,600	600	0.0013		6
5		TCAA	200	10	0.5	2,800 g L^{-1} Fe^0	7
6		DCAA	40	10	0.4	900 g L^{-1} BAC [a]	7
7		MCAA	76	10	0.4	900 g L^{-1} BAC [a]	7
8		DCAA	5,805	4500	0.0003	2.4 g L^{-1} Fe^0	8
9		BAA	9,710	10	0.006		8
10		DBAA	9,803	15	0.14		8
11		TBAA	4,451	10	0.34		8
12	Photochemical system	MCAA	189	3	0.12	1.0 mM S(IV)	9
13		MCAA	4,253	3	0.061	3.73 × 10^{-6} einstein L^{-1} s^{-1} UV	9
14		MCAA	20, 000	50	3.996	3.13 × 10^4 μmol m^{-2} s^{-1} UV	10
15		DCAA	20, 000	30	9.738		10
16		TCAA	20, 000	25	13.902		10
17		TCAA	1, 000	90	0.042	0.34 g L^{-1} F-TiO_2 [b] 12 V/V% MeOH [c] 87 μW cm^{-2} UV	11
18	Electrocatalysis	DBAA	1, 000	0.183	9.16	-1.5 V/SHE using REM [d]	12
19		DBAA	1, 000	0.183	33.3	-1.5 V/SHE [e] on WCNT-REM [d]	12
20		DBAA	1, 000	0.183	18.3	-1.5 V/SHE using PAC-REM [d]	12
21		TCAA	500	40	0.041	-0.5 V using Pd/rGO/CFP [f]	13
22		TCAA	500	20	0.137	-1.2 V/SCE [g] on the GR−Cu foam [h]	14
23		TCAA	500	30	0.0613	0.9 mA cm^{-2} over	15
24		TCAA	500	10	0.5802	Pd-In/Al_2O_3	15

25		TCAA	500	100	0.0283	-1.2 V/SCE over ANP electrode [i]	16
26		TCAA	500	100	0.0184	-1.2 V/SCE on Pd/C electrode	16
27		TCAA	5, 000	60	1.48	-0.5 V/RHE on	17
28		TCAA	5, 000	60	0.091	CCC/Pd [j] with visible light	17
29		TCAA	500	10	0.271	-1.2 V at NG-Cu foam cathode [k]	18
30		TCAA	817, 000	150	0.0087	70 mA on the Pd-loaded Ni cathode	19
**31**		**DCAA**	**180**	**6.66667E-05**	**51000**	**1.0 mM S(IV) Fe/GO membrane**	**This work**

a. BAC indicates biologically active carbon processes.

b. F-TiO_2 represents doping fluoride (F) on TiO_2.

c. V/V indicates the volume ratio of methanol over water.

d. REM, MWCNT-REM, and PAC-REM represent Ti_4O_7 reactive electrochemical membranes, multiwalled carbon nanotubes-Ti_4O_7 composite reactive electrochemical membranes, and powder activated carbon-Ti_4O_7 composite reactive electrochemical membranes, respectively.

e. SHE indicates a standard hydrogen electrode.

f. Pd/rGO/CFP indicates a Pd/reduced graphene oxide hybrid catalyst fabricated on carbon fiber paper.

g. SCE represents a saturated calomel electrode.

h. GR−Cu foam represents a three-dimensional graphene−copper (3D GR−Cu) foam electrode.

i. ANP indicates amorphous nickel phosphide.

j. CCC/Pd represents a Cu/Cu_2O/CuO electrode.

k. NG-Cu foam represents a noble metal-free N-doped graphene-Cu (NG-Cu) foam electrode.

Supplementary Table 2. Comparison of permeance and removal efficiency between our Fe/GO membrane and other nanofiltration and reverse osmosis membranes.

No.	Processes	Conc. (μg L^{-1})	Flux (LMH bar^{-1})	Rejection (%)	Ref.
1	RO [a]	100	7.25	90	[20]
2	NF [b]	100	7.69	90	[20]
3	NF	100	16.1	60	[20]
4	RO	100	2.18	90	[20]
5	NF	100	7.83	91	[21]
6	NF	100	7.83	94	[21]
7	RO	100	9.22	95	[21]
8	RO	100	9.22	96	[21]
9	FO [c]	200	0.86	94.6	[22]
10	FO	200	0.86	73.8	[22]
11	FO	200	0.86	89.1	[22]
12	FO	200	0.86	97	[22]
13	RO	50	4.9	98	[23]
14	RO	50	4.9	74.6	[23]
15	NF	100	7.69	95	[24]
16	NF	100	16.1	85	[24]
17	NF	100	16.1	90	[24]
18	NF	100	16.1	95	[24]
19	NF		22.8	75	[25]
20	NF		25	92	[25]
21	NF		25	81.2	[25]
22	NF		8	98	[26]
23	NF		16.9	75	[26]
24	NF		16.9	48	[26]

25	**DCAA**	**180**	**48.6**	**96.8**	**This work**

a, b, and c separately indicate reverse osmosis, nanofiltration, and forward osmosis.

Supplementary Table 3. Weight ratios in entire membrane system and confined reaction region.

Nominal ratios in entire membrane system [a]					
Content (g)		Mass ratio ($g\ g^{-1}$)		Molar ratio ($mol\ mol^{-1}$)	
Fe	1.30×10^{-5}				
Sulfite	8.00×10^{-2}	Fe to sulfite	1.62×10^{-4}	Fe to sulfite	2.34×10^{-4}
DCAA	1.80×10^{-4}	Fe to DCAA	7.24×10^{-2}	Fe to DCAA	1.68×10^{-1}
Effective ratios in confined reaction region					
Content (g)		Mass ratio ($g\ g^{-1}$)		Molar ratio ($mol\ mol^{-1}$)	
Fe	1.30×10^{-5}				
GO	4.00×10^{-4}				
Water	$(4.00–16.0) \times 10^{-4}$ [b]				
Sulfite	$(1.60–6.40) \times 10^{-8}$ [c]	Fe to sulfite	815–204	Fe to sulfite	1168–292
DCAA	$(7.20–28.8) \times 10^{-11}$ [d]	Fe to DCAA	181226–45306	Fe to DCAA	418963–104741

a. For a membrane coupon area of 55.4 cm^2.

b. Based on a water uptake of 1–4 $g\ g^{-1}$ GO.[5]

c. Sulfite content in the reaction region is calculated based on the water uptake by GO and its effective concentration in the catalytic membrane. For sulfite with a rejection of 50% by the membrane, its effective concentration in the reaction region is estimated as half of the bulk concentration, i.e., (100%-50%) × 1 mM = 0.5 mM.

d. DCAA content in the reaction region is calculated based on the water uptake by GO and its effective concentration in the catalytic membrane. Since the rejection of DCAA by the membrane is negligible (< 2%), its effective concentration in the reaction region is approximated by the bulk concentration (180 $\mu g\ L^{-1}$).

Supplementary References

1. Xiao, Q. & Yu, S. Reduction of bromate from drinking water by sulfite/ferric ion systems: Efficacy and mechanisms. *J Hazard Mater* **418**, 125940 (2021).

2. Xiao, Q. & Yu, S. The role of dissolved oxygen in the sulfite/divalent transition metal ion system: degradation performances and mechanisms. *Chem Eng J* **417**, 129115 (2021).

3. Dong, H., Wei, G., Yin, D. & Guan, X. Mechanistic insight into the generation of reactive oxygen species in sulfite activation with Fe(III) for contaminants degradation. *J Hazard Mater* **384**, 121497 (2020).

4. Dong, H., *et al.* Reinvestigating the role of reactive species in the oxidation of organic co-contaminants during Cr(VI) reactions with sulfite. *Chemosphere* **196**, 593-597 (2018).

5. Chen, L., *et al.* Ion sieving in graphene oxide membranes via cationic control of interlayer spacing. *Nature* **550**, 380-383 (2017).

6. Hozalski, R. M., Zhang, L. & Arnold, W. A. Reduction of Haloacetic Acids by Fe0: Implications for Treatment and Fate. *Environ Sci Technol* **35**, 2258-2263 (2001).

7. Tang, S., Wang, X. M., Yang, H. W. & Xie, Y. F. Haloacetic acid removal by sequential zero-valent iron reduction and biologically active carbon degradation. *Chemosphere* **90**, 1563-1567 (2013).

8. Zhang, L., Arnold, W. A. & Hozalski, R. M. Kinetics of Haloacetic Acid Reactions with Fe(0). *Environ Sci Technol* **38**, 6881-6889 (2004).

9. Li, X., *et al.* Efficient reductive dechlorination of monochloroacetic acid by sulfite/UV process. *Environ Sci Technol* **46**, 7342-7349 (2012).

10. Zhang, L., Ai, W., Li, C., Zhang, Q. & Li, T. Efficient photolytic degradation of disinfection by-products by using a high photon flux UV system: monochloroacetic acid, dichloroacetic acid and trichloroacetic acid. *Water Supply* **18**, 2063-2070 (2018).

11. Massoudinejad, M., Yazdanbakhsh, A., Amini, M. M., Nourmoradi, H. & Keramati, H. Enhanced photocatalytic reduction of trichloroacetic acid using F-TiO_2 in the presence of methanol: degradation

kinetics and byproducts pathway. *International Journal of Environmental Analytical Chemistry* **102**, 2461-2482 (2020).

12. Almassi, S., Samonte, P. R. V., Li, Z., Xu, W. & Chaplin, B. P. Mechanistic Investigation of Haloacetic Acid Reduction Using Carbon-Ti_4O_7 Composite Reactive Electrochemical Membranes. *Environ Sci Technol* **54**, 1982-1991 (2020).

13. Mao, R., *et al.* Enhanced indirect atomic H* reduction at a hybrid Pd/graphene cathode for electrochemical dechlorination under low negative potentials. *Environmental Science: Nano* **5**, 2282-2292 (2018).

14. Mao, R., *et al.* Dechlorination of Trichloroacetic Acid Using a Noble Metal-Free Graphene-Cu Foam Electrode via Direct Cathodic Reduction and Atomic H. *Environ Sci Technol* **50**, 3829-3837 (2016).

15. Liu, Y., *et al.* Reductive dechlorination of trichloroacetic acid (TCAA) by electrochemical process over Pd-In/Al_2O_3 catalyst. *Electrochimica Acta* **232**, 13-21 (2017).

16. Yao, Q., *et al.* Amorphous nickel phosphide as a noble metal-free cathode for electrochemical dechlorination. *Water Res* **165**, 114930 (2019).

17. Zhang, J., *et al.* Synchronous Reduction-Oxidation Process for Efficient Removal of Trichloroacetic Acid: H* Initiates Dechlorination and .OH Is Responsible for Removal Efficiency. *Environ Sci Technol* **53**, 14586-14594 (2019).

18. Mao, R., *et al.* Insights into co-removal of trichloroacetic acid and bromate by an electroreduction process: Competitive reaction mechanism and enhanced atomic H* stabilization. *Chemical Engineering Journal* **429**, (2022).

19. Zhao, C., *et al.* Simultaneous Degradation of Aqueous Trichloroacetic Acid by the Combined Action of Anodic Contact Glow Discharge Electrolysis and Normal Electrolytic Processes at the Cathode. *Plasma Chemistry and Plasma Processing* **39**, 751-767 (2019).

20. Yang, L., *et al.* Removal of haloacetic acids from swimming pool water by reverse osmosis and nanofiltration. *Water Res* **116**, 116-125 (2017).

21. Kimura, K., *et al.* Rejection of organic micropollutants (disinfection by-products, endocrine disrupting compounds, and pharmaceutically active compounds) by NF/RO membranes. *Journal of Membrane Science* **227**, 113-121 (2003).

22. Kong, F.-X., Yang, H.-W., Wang, X.-M. & Xie, Y. F. Rejection of nine haloacetic acids and coupled reverse draw solute permeation in forward osmosis. *Desalination* **341**, 1-9 (2014).

23. Wang, L., Sun, Y. & Chen, B. Rejection of haloacetic acids in water by multi-stage reverse osmosis: Efficiency, mechanisms, and influencing factors. *Water Res* **144**, 383-392 (2018).

24. Yang, L., *et al.* Role of calcium ions on the removal of haloacetic acids from swimming pool water by nanofiltration: mechanisms and implications. *Water Res* **110**, 332-341 (2017).

25. Chellam, S. Effects of Nanofiltration on Trihalomethane and Haloacetic Acid Precursor Removal and Speciation in Waters Containing Low Concentrations of Bromide Ion. *Environ Sci Technol* **34**, 1813-1820 (2000).

26. Zhao, H., *et al.* Magnesium-Induced Variation of Polyamide Membrane Behavior for the Treatment of Haloacetic Acids in Swimming Pool Waters. *ACS ES&T Water* **1**, 346-355 (2020).
